# Age and viral replication, not vertical HIV acquisition, drive inflammation and T-cell dysfunction in heavily treatment experienced: data from the Prestigio Registry

**DOI:** 10.1097/QAD.0000000000004415

**Published:** 2026-03-26

**Authors:** Valeria Bono, Camilla Tincati, Matteo Augello, Roberta Rovito, Vincenzo Spagnuolo, Laura Galli, Antonio di Biagio, Elisa Garlassi, Maria Cristina Moioli, Emanuele Focà, Antonella Castagna, Giulia Marchetti

**Affiliations:** a Clinic of Infectious Diseases and Tropical Medicine, San Paolo Hospital, ASST Santi Paolo e Carlo, Department of Health Sciences; b Infectious Diseases, IRCCS San Raffaele Scientific Institute, Vita-Salute San Raffaele, University of Milan, Milan; c Department of Infectious Disease, IRCCS AOU San Martino IST, (DISSAL), University of Genoa; d Malattie Infettive Arcispedale S. Maria Nuova-IRCSS, Reggio Emilia; e Department of Infectious Diseases, ASST Grande Ospedale Metropolitano Niguarda, Milan; f Division of Infectious and Tropical Diseases, ASST Spedali Civili Hospital, University of Brescia, Brescia, Italy.

## Abstract

Heavily treatment-experienced (HTE) individuals carry a high inflammatory burden, which is potentially increased in those with vertical HIV transmission (VT), due to lifelong viral exposure. We assessed inflammation and T-cell activation/exhaustion/senescence in HTE with VT or horizontal transmission (HT). Interleukin-6 (IL-6) was lower in VT and positively correlated with age. T-cell dysfunction was greater in HTE than people without HIV (PWOH), mainly driven by viremic VT and HT. Age and viral replication, rather than transmission mode, underlie immune dysregulation in HTE.

HTE people with HIV (PWH) are defined as those who harbour a multidrug resistant virus [1]. Antiretroviral resistance to multiple drug classes is strictly dependent on suboptimal treatment exposure which may lead to active HIV replication [2].

Lack of viral control has been independently associated with high levels of inflammation [3] which is an established cause of clinical progression [4]. Of note, residual inflammation persists even in the setting of viral suppression and is linked to the development of noninfectious comorbidities [5].

In keeping with these findings, recent data from our group data have demonstrated that plasma inflammatory burden follows a hierarchical distribution, with the highest levels observed in viremic HTE individuals, intermediate levels in aviremic HTE, and the lowest levels in virologically suppressed PWH without evidence of drug resistance [6]. These results highlight an excess risk of noninfectious comorbidities in HTE, regardless HIV RNA load.

PWH with vertical transmission (VT) are known to experience adherence challenges increasing their risk of developing antiretroviral drug resistance [7,8]. Further, life-long exposure to HIV in this population may fuel systemic inflammation [9] and contribute to the pathogenesis of comorbidities at a younger age [10].

Taken together, these observations lead us to hypothesize that HTE with vertical HIV acquisition feature higher inflammation and T-cell dysfunction than HTE with horizontal transmission (HT).

HTE individuals with either VT and HT were enrolled from the Prestigio Registry [11]. All had documented resistance to nucleoside reverse transcriptase inhibitors, non-nucleoside reverse transcriptase inhibitors, protease inhibitors, and/or integrase strand transfer inhibitors. Participants were further classified according to virological status at sampling as suppressed (viral load, VL < 50 cp/ml) or viremic (VL > 200 cp/ml), as illustrated in Fig. 1a. Demographic and clinical characteristics of the study population were recorded. Propensity score was used to match VT and HT for sex, HIV duration, CD4^+^ T-cell nadir and HIV RNA load at plasma sampling.

**Fig. 1:**
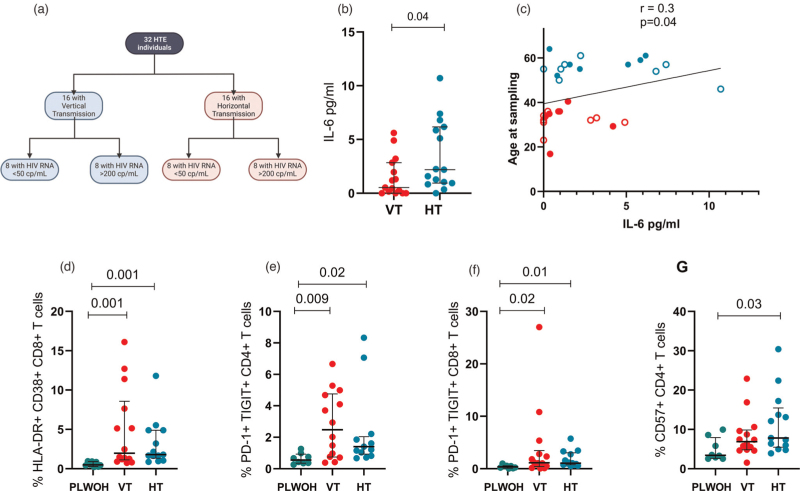
Immunological and inflammatory profiles in HIV transmission groups.

(a) Schematic representation of the study cohort including 32 HIV transmission-exposed (HTE) individuals divided into two groups based on the route of HIV acquisition: vertical transmission (VT, *n* = 16) and horizontal transmission (HT, *n* = 16). Each group was further stratified by HIV RNA levels (<50 or >200 copies/ml). (b) Plasma IL-6 concentrations (pg/ml) in VT and HT individuals. Each dot represents one individual; lines indicate median and interquartile range. Statistical significance was determined using the Mann–Whitney *U* test. (c) Correlation between plasma IL-6 levels and age at sampling in HTE individuals. Spearman's correlation coefficient (*r*) and *p*-value are shown. (d–g) Frequency of activation, exhaustion and senescence markers on CD4^+^ and CD8^+^ T-cells in VT, and HT groups compared to PWOH. Statistical analysis: Kruskal–Wallis test, multiple comparisons.

A broad range of plasma cytokines and chemokines [GM-CSF, interferon gamma (IFN-α), IFN-γ, interleukin (IL)-2, IL-4, IL-5, IL-6, IL-9, IL-10, IL-12p70, IL-17A, tumour necrosis factor alpha (TNF-α)] was assessed by cytometric bead array; sCD14 was measured by ELISA samples, following the manufacturer's instructions.

T-cell dysfunction was measured by the surface expression of senescence (CD57), activation (HLA-DR/CD38) and exhaustion (PD-1/TIGIT) markers on CD4^+^ and CD8^+^ T-cells using flow cytometry. Eight people without HIV (PWOH), matched for age and sex, were included as controls to provide a baseline for comparison. They were selected to reflect the age distribution of both study groups (four matched to the younger VT group, median 31 years, and four to the older HT group, median 50 years). Overall, the age of the control group did not differ significantly from either PWH group (*P* = 0.3).

Kruskal–Wallis and Mann–Whitney tests were used for comparisons. Fisher's exact test for categorical variables (expressed as percentages). Spearman's correlation was used to correlate age at sampling and inflammation markers. Data were analysed with GraphPad Prism 10.2.1.

Sixteen VT and 16 HT individuals were enrolled (Table S1, Supplemental Digital Content). In each group, 8/16 had undetectable viral load (HIV RNA < 50 copies/ml) and 8/16 had detectable viral load (HIV RNA > 200 copies/ml). 62.5% were female in both groups. VT were younger than HT individuals (31 years, IQR 27–33 vs. 56, IQR 54–59; *P* < 0.0001), yet duration of HIV infection was comparable in the two groups (31 years, IQR 27–33 vs. 30 years, IQR 25–32; *P* = 0.7). The total duration of virologic suppression (VL < 50 cp/ml; *P* = 0.7) and detectable viremia (VL > 200 cp/ml; *P* = 0.8), expressed in years, was similar between VT and HT individuals (Table S1, Supplemental Digital Content). Among comorbidities, hypertension was more frequent in individuals with horizontal HIV transmission compared to those with vertical transmission (Table S1, Supplemental Digital Content). No statistically significant difference was observed in CD4^+^ T-cell nadir and absolute count at the time of sampling between VT and HT.

VT showed lower IL-6 than HT (0.526 pg/ml, IQR 0–2.85 vs. 2.198 pg/ml, IQR 0.94–6.17; *P* = 0.04, Fig. 1b). However, when stratifying according to viremia, VT and HT did not show significant differences in IL-6 levels. The remaining cytokines showed comparable levels between groups, except for a trend to lower IL-10 in VT overall (*P* = 0.08) and in those with HIV RNA > 200 cp/ml (0 pg/ml, IQR 0–0 vs. 13.5 pg/ml, IQR 0–28.1; *P* = 0.06).

No significant correlations were observed between plasma cytokines and demographic or viro-immunologic parameters, except for IL-6, which was positively associated with age (*r* = 0.362, *P* = 0.04, Fig. 1c).

No differences were detected between VT and HT in terms of CD4^+^ and CD8^+^ T-cell dysfunction. However, compared to PWOH, VT and HT displayed higher activated CD8^+^HLA-DR^+^CD38^+^ (PWOH: 0.4%, IQR 0.2–0.9; VT: 1.9%, IQR 1.1–8.5; *P* = 0.001; HT: 1.7%, IQR 1.3–4.8; *P* = 0.001, Fig. 1d), exhausted PD-1^+^TIGIT^+^ CD4^+^ (PWOH: 0.5%, IQR 0.3–0.9; VT: 2.4%, IQR 0.7–4.7; *P* = 0.009; HT: 1.4%, IQR 0.9–2; *P* = 0.02, Fig. 1e) and CD8^+^ (PWOH: 0.3%, IQR 0.1–0.5; VT: 1.1%, IQR = 0.4–3.4; *P* = 0.02; HT: 1%, IQR 0.7–3; *P* = 0.01, Fig. 1f). Similarly, senescent CD57^+^CD4^+^ T-cells were increased in HT alone compared to PWOH (7.8%, IQR 5.4–15.4 vs. 3.4%, IQR 2.6–7.9; *P* = 0.03. Fig. 1g). These findings were retained in the viremic subgroups of VT and HT who showed higher frequencies of CD4^+^ and CD8^+^ T-cells with an activated HLA-DR^+^CD38^+^ (Fig. S1A, Supplemental Digital Content; Fig. S1B, Supplemental Digital Content) and exhausted PD-1^+^TIGIT^+^ phenotype (Fig. S1C, Supplemental Digital Content; Fig. S1D, Supplemental Digital Content) compared to PWOH. In contrast, in the virally-suppressed cohort, only HT displayed higher CD8+ cells with an activated (1.6% IQR 0.9–1.7 vs. 0.4% IQR 0.2–0.9, *P* = 0.003, Fig. S1E, Supplemental Digital Content) and exhausted phenotype (1% IQR 0.7–1.2 vs. 0.3%, IQR 0.1–0.5, *P* = 0.04, Fig. S1F, Supplemental Digital Content) compared to PWOH. When stratifying PWOH controls by age, T-cell dysfunction markers were significantly lower in both younger and older PWOH compared to VT and HT, respectively, confirming that PWH display higher levels of T-cell dysfunction overall (Fig. S1G–L, Supplemental Digital Content).

No link was observed between T-cell phenotype and demographic or viro-immunologic parameters.

In line with previous studies showing that age has a central role in driving inflammation in treated PWH [12,13], our findings suggest that in the context of HTE, aging rather than the mode of HIV acquisition is closely linked to inflammation. The VT group was substantially younger than the HT group, which likely contributes to some of the observed differences in inflammatory markers. IL-6 emerged as the most robust age-associated marker, consistent with the concept of inflammaging, while TNF-α showed only nonsignificant trends in older HT individuals, likely reflecting inter-individual variability in baseline plasma levels as previously described [13]. These observations highlight that age should be carefully considered when interpreting differences between VT and HT groups. VT and HT individuals were matched for duration of HIV infection (median 30 years in both groups; Table S1, Supplemental Digital Content). While age appears to be the main driver of inflammation in this cohort, long-term HIV exposure may also contribute to the inflammatory burden, suggesting that both aging and cumulative infection could influence immune activation in heavily treatment-experienced individuals.

Of note, although T-cell dysfunction in HTE was mainly driven by uncontrolled viremia, highlighting the key role of viral replication in T-cell homeostatic imbalances [9], aviremic HT showed higher activated HLA-DR^+^CD38^+^ and exhausted PD-1^+^TIGIT^+^ CD8^+^T-cells, which may also mirror their older age.

A limitation of our study is the age difference between the VT and HT groups, which could partially influence some inflammatory marker. Further, our data on T-cell dysfunction in HTE should be detailed in the context of VT and HT in virally-suppressed PWH with no history of viral failure.

Taken together, the present work shows that HTE with vertical HIV acquisition do not feature greater immune dysregulation than HTE with horizontal transmission. Given the key role of age and viral replication in driving inflammatory imbalances in the context of multidrug resistance, our findings highlight the need of complete HIV suppression and careful management of noninfectious comorbidities in HTE of all ages.

## Acknowledgements

**Presented in part**: 12th IAS Conference on HIV Science, July 23–26 2023, Brisbane, Australia.

### Conflicts of interest

There are no conflicts of interest.

## Supplementary Material

Supplemental Digital Content
