# Supplementary material for: Age and viral replication, not vertical HIV acquisition, drive inflammation and T-cell dysfunction in heavily treatment experienced: data from the Prestigio Registry
Source: AIDS. 2026 Mar 26;40(5):690–3. doi: 10.1097/QAD.0000000000004415 (PMC13034735; doi:10.1097/QAD.0000000000004415)

**Table S1. Demographic and clinical features of the study population**

| \|  \| VT  (N=16) \| HT  (N=16) \| p-value \| \| \| --- \| --- \| --- \| --- \| --- \| \| Age at sampling, (years), *median (IQR)* \| 31 (27-33) \| 56 (54-59) \| **<0.0001** \| \| Sex female, *n (%)* \| 10 (62.5) \| 10 (62.5) \| 1.000 \| \| Ethnicity, *n (%)*  Caucasian \| 16 (100) \| 16 (100) \| 1.000 \| \| Alcohol use, *n (%)*  Yes  No  Unknown \| 4 (25)  7 (43.7)  5 (31.2) \| 5 (31.2)  3 (18.7)  7 (43.7) \| 0.3 \| \| Smokers, *n (%)*  Yes  No  Unknown \| 6 (37.5)  6 (37.5)  4 (25) \| 10 (62.5)  3 (18.7)  3 (18.7) \| 0.4 \| \| Drug abuse, *n (%)*  Yes  No  Unknown \| 0 (0)  13 (81.2)  3 (18.7) \| 0 (0)  11 (68.7)  5 (31.2) \| 0.5 \| \| Socioeconomic status, *n (%)*  Employed  Unemployed  Unknown \| 6 (37.5)  2 (12.5)  8 (50) \| 6 (37.5)  4 (25)  6 (37.5) \| 0.7 \| \| Comorbidities, *(n, %)*  Cardiovascular disease  Chronic kidney disease  Liver disease  Diabetes  Hypertension  History of cancer  AIDS - C events \| 0 (0%)  0 (0%)  1 (6.2%)  0 (0%)  0 (0%)  2 (12.5%)  7 (43.7%) \| 1 (6.2%)  1 (6.2%)  1 (6.2%)  1 (6.2%)  6 (37.5%)  4 (25%)  2 (12.5%) \| 1.000  1.000  1.000  1.000  **0.01**  0.6  0.1 \| \| BMI, *n (%)*  <25 kg/m^2^  25–30 kg/m^2^  >30 kg/m^2^ \| 0 (0%)  0 (0%)  6 (37.5%) \| 0 (0%)  0 (0%)  7 (43.7%) \| 1.000 \| \| Time since HIV diagnosis, (years), *median (IQR)* \| 31 (27-33) \| 30 (25-32) \| 0.7 \| \| Total time with VL <50 cp/mL, (years), *median (IQR)* \| 1 (0.5-5.7) \| 1.9 (0.2-6.8) \| 0.7 \| \| Total time with VL >200 cp/mL, (years), *median (IQR)* \| 5 (1.6-7.9) \| 4.9 (4-6) \| 0.8 \| \| CD4+ nadir, (cells/mm^3^), *median (IQR)* \| 90 (16-186) \| 127 (21-200) \| 0.7 \| \| CD4+ at sampling, (cells/mm^3^), *median (IQR)* \| 394 (179-1011) \| 547 (149-770) \| 0.5 \| \| CD8+ at sampling, (cells/mm^3^), *median (IQR)* \| 806 (571-1117) \| 672 (510-949) \| 0.4 \| \| HIV-RNA, copies/mL, median (IQR) \| 365.5 (5.6-41405) \| 141 (36.7-4098) \| 0.9 \| \| HIV-RNA at sampling, *n (%)*  <50 copies/mL  >200 copies/mL \| 8 (50%)  8 (50%) \| 8 (50%)  8 (50%) \| 1.000 \| \| HBsAg, *n (%)* \| 0 (0%) \| 0 (0%) \| 1.000 \| \| HCV-Ab positive, *n (%)* \| 0 (0%) \| 4 (25%) \| 0.1 \| |
| --- | --- | --- | --- | --- | --- | --- | --- | --- | --- | --- | --- | --- | --- | --- | --- | --- | --- | --- | --- | --- | --- | --- | --- | --- | --- | --- | --- | --- | --- | --- | --- | --- | --- | --- | --- | --- | --- | --- | --- | --- | --- | --- | --- | --- | --- | --- | --- | --- | --- | --- | --- | --- | --- | --- | --- | --- | --- | --- | --- | --- | --- | --- | --- | --- | --- | --- | --- | --- | --- | --- | --- | --- | --- | --- | --- | --- | --- | --- | --- | --- | --- |

**Legend**: VT: vertical transmission; HT: horizontal transmission; IQR: interquartile range; Statistical analyses, Mann-Whitney U test, Fisher exact test, as appropriate.

**Figure S1. Immune dysfunction in HTE with and without vertical transmission, according to viral load.** Frequencies of activated and exhausted CD4+ and CD8+ T-cells in VT and HT individuals and 8 people living without HIV, matched for age/sex. (**A-F**). Frequencies of activated, exhausted, and senescent CD4+ and CD8+ T-cells in VT and HT individuals, compared to PLWOH stratified into younger and older subgroups to match the age distribution of VT and HT groups (**G-L**). S*tatistical analysis*: Kruskal-Wallis test, Multiple Comparisons.


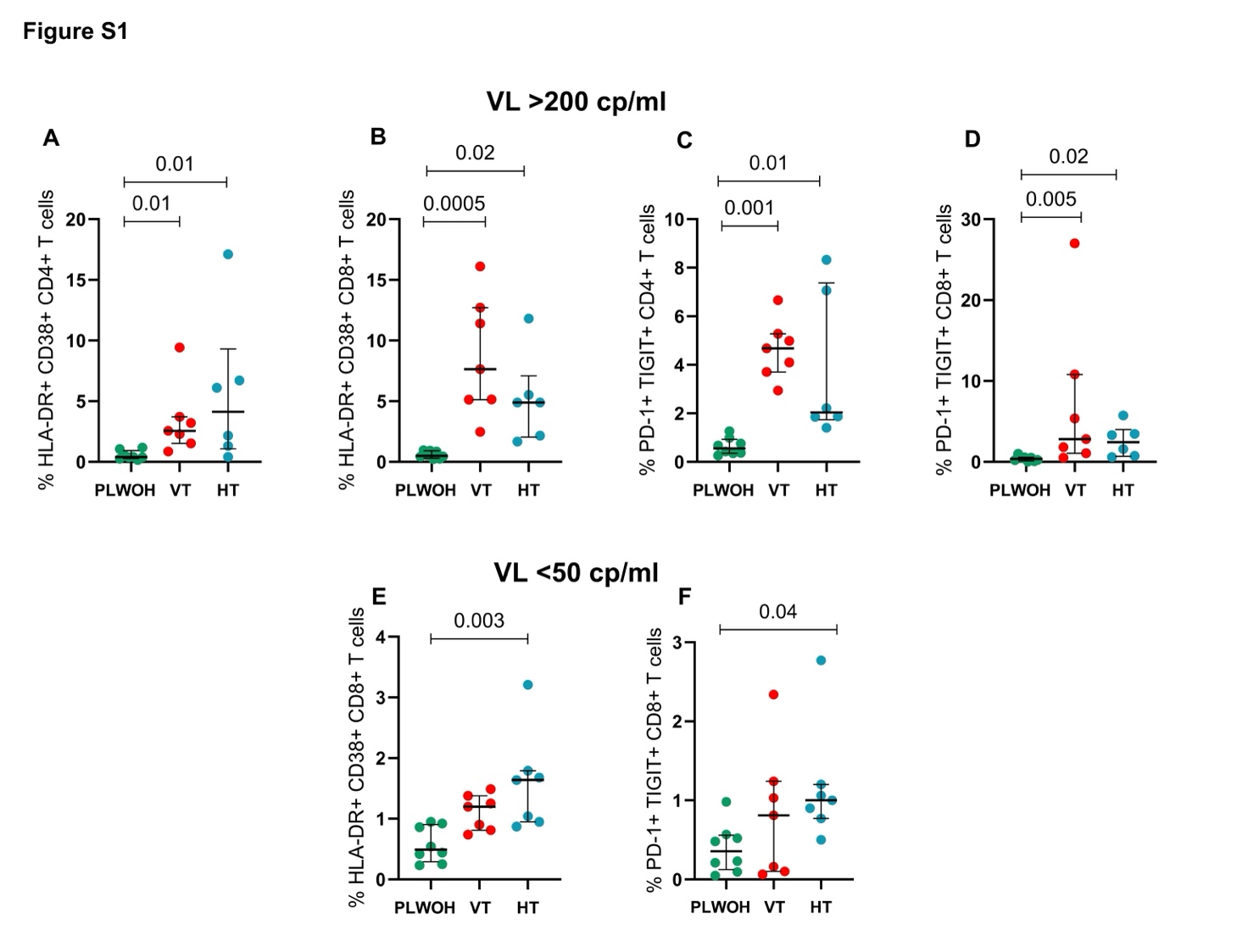


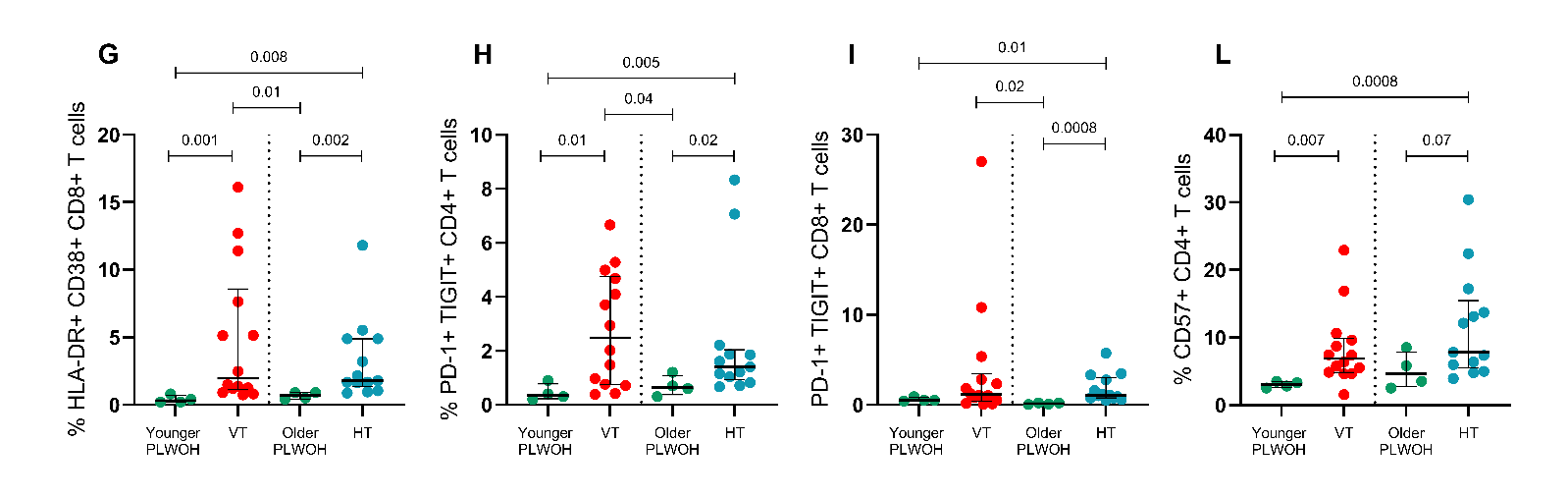

Supplement: Supplemental Digital Content [file aids-40-690-s001.docx]
